# Supplementary material for: OpenMM 7: Rapid development of high performance algorithms for molecular dynamics
Source: PLoS Comput Biol. 2017 Jul 26;13(7):e1005659. doi: 10.1371/journal.pcbi.1005659 (PMC5549999; doi:10.1371/journal.pcbi.1005659)
Supplement: S1 Source Code — (ZIP) [file pcbi.1005659.s002.zip › OpenMM-7.0.1-Source/docs/api-c++/generated/OpenMM.LocalCoordinatesSite.html]

LocalCoordinatesSite — OpenMM @OPENMM\_MAJOR\_VERSION@.@OPENMM\_MINOR\_VERSION@ documentation


# LocalCoordinatesSite¶

*class* `OpenMM::``LocalCoordinatesSite`¶
:   This is a `VirtualSite` that uses the locations of three other particles to compute a local coordinate system, then places the virtual site at a fixed location in that coordinate system. The origin of the coordinate system and the directions of its x and y axes are each specified as a weighted sum of the locations of the three particles:

    origin = w_1_ r_1_ + w_2_ r_2_ + w_3_ r_3_ None

    xdir = w_1_ r_1_ + w_2_ r_2_ + w_3_ r_3_ None

    ydir = w_1_ r_1_ + w_2_ r_2_ + w_3_ r_3_ None

    For the origin, the three weights must add to one. For example if (w_1_ , w_2_ , w_3_ ) = (1.0, 0.0, 0.0), the origin of the local coordinate system is at the location of particle 1. For xdir and ydir, the weights must add to zero. For excample, if (w_1_ , w_2_ , w_3_ ) = (-1.0, 0.5, 0.5), the x axis points from particle 1 toward the midpoint between particles 2 and 3.

    The z direction is computed as zdir = xdir x ydir. To ensure the axes are all orthogonal, ydir is then recomputed as ydir = zdir x xdir. All three axis vectors are then normalized, and the virtual site location is set to

    origin + x\*xdir + y\*ydir + z\*zdir

    Methods

    |  |  |
    | --- | --- |
    | `LocalCoordinatesSite` | Create a new `LocalCoordinatesSite` virtual site. |
    | `getOriginWeights` | Get the weight factors for the three particles when computing the origin location. |
    | `getXWeights` | Get the weight factors for the three particles when computing xdir. |
    | `getYWeights` | Get the weight factors for the three particles when computing ydir. |
    | `getLocalPosition` | Get the position of the virtual site in the local coordinate system. |

    `LocalCoordinatesSite`(int *particle1*, int *particle2*, int *particle3*, *const* Vec3 &*originWeights*, *const* Vec3 &*xWeights*, *const* Vec3 &*yWeights*, *const* Vec3 &*localPosition*)¶
    :   Create a new `LocalCoordinatesSite` virtual site.

        |  |  |
        | --- | --- |
        | Parameters: |  |

        - **particle1** – the index of the first particle
        - **particle2** – the index of the second particle
        - **particle3** – the index of the third particle
        - **originWeights** – the weight factors for the three particles when computing the origin location
        - **xWeights** – the weight factors for the three particles when computing xdir
        - **yWeights** – the weight factors for the three particles when computing ydir
        - **localPosition** – the position of the virtual site in the local coordinate system

    *const* Vec3 &`getOriginWeights`() *const*¶
    :   Get the weight factors for the three particles when computing the origin location.

    *const* Vec3 &`getXWeights`() *const*¶
    :   Get the weight factors for the three particles when computing xdir.

    *const* Vec3 &`getYWeights`() *const*¶
    :   Get the weight factors for the three particles when computing ydir.

    *const* Vec3 &`getLocalPosition`() *const*¶
    :   Get the position of the virtual site in the local coordinate system.

High performance molecular simulation on GPUs


### Navigation

- Core Objects
- Forces
- Integrators
- Extras

©2015, Stanford University and the Authors.
|
Powered by Sphinx 1.3.1
& Alabaster 0.7.7
|
Page source
